# Supplementary material for: Identification and Preliminary Clinical Validation of Key Extracellular Proteins as the Potential Biomarkers in Hashimoto’s Thyroiditis by Comprehensive Analysis
Source: Biomedicines. 2023 Nov 24;11(12):3127. doi: 10.3390/biomedicines11123127 (PMC10740579; doi:10.3390/biomedicines11123127)
Supplement: Supplementary file 1 [file biomedicines-11-03127-s001.zip › Supplementary Table S1.pdf]

Supplementary Table S1. Details of the PPI network of EP-DEGs constructed by the STRING database.

#node1	node2	node1_string_id	node2_string_id	neighborhood_on_chromosome	gene_fusion	phylogenetic_cooccurrence	homology	coexpression	experimentally_determined_interaction	database_annotated	automated_textmining	combined_score
ADAMDEC1	CXCL9	9606.ENSP00000256412	9606.ENSP00000354901	0	0	0	0	0.335	0	0	0.266	0.491
ADAMDEC1	CXCL13	9606.ENSP00000256412	9606.ENSP00000286758	0	0	0	0	0.492	0	0	0.242	0.598
APOC1	SAA2	9606.ENSP00000465356	9606.ENSP00000436126	0	0	0	0	0.049	0	0.54	0.407	0.717
CCL5	GZMK	9606.ENSP00000474412	9606.ENSP00000231009	0	0	0	0	0.561	0	0	0.675	0.851
CCL5	LY96	9606.ENSP00000474412	9606.ENSP00000284818	0	0	0	0	0.064	0	0	0.434	0.448
CCL5	CXCL13	9606.ENSP00000474412	9606.ENSP00000286758	0	0	0	0	0.144	0	0.5	0.905	0.956
CCL5	CXCL10	9606.ENSP00000474412	9606.ENSP00000305651	0	0	0	0	0.362	0.994	0.5	0.95	0.999
CCL5	CXCL11	9606.ENSP00000474412	9606.ENSP00000306884	0	0	0	0	0.355	0.994	0.5	0.885	0.999
CCL5	CXCL9	9606.ENSP00000474412	9606.ENSP00000354901	0	0	0	0	0.371	0.994	0.5	0.948	0.999
CCL5	XCL1	9606.ENSP00000474412	9606.ENSP00000356792	0	0	0	0.803	0.215	0.994	0	0.285	0.996
CCL5	SAA2	9606.ENSP00000474412	9606.ENSP00000436126	0	0	0	0	0.074	0	0	0.657	0.669
CXCL10	GZMK	9606.ENSP00000305651	9606.ENSP00000231009	0	0	0	0	0.149	0	0	0.355	0.428
CXCL10	LY96	9606.ENSP00000305651	9606.ENSP00000284818	0	0	0	0	0.126	0	0	0.418	0.469
CXCL10	CXCL13	9606.ENSP00000305651	9606.ENSP00000286758	0	0	0	0	0.201	0	0.9	0.939	0.994
CXCL10	SAA2	9606.ENSP00000305651	9606.ENSP00000436126	0	0	0	0	0.056	0	0	0.777	0.78
CXCL10	XCL1	9606.ENSP00000305651	9606.ENSP00000356792	0	0	0	0	0.19	0.994	0	0.613	0.997
CXCL10	CXCL9	9606.ENSP00000305651	9606.ENSP00000354901	0	0	0	0.856	0.836	0.994	0.9	0.444	0.999
CXCL10	CXCL11	9606.ENSP00000305651	9606.ENSP00000306884	0	0	0	0.811	0.824	0.994	0.9	0.467	0.999
CXCL11	CXCL13	9606.ENSP00000306884	9606.ENSP00000286758	0	0	0	0	0.262	0	0.9	0.839	0.987
CXCL11	SAA2	9606.ENSP00000306884	9606.ENSP00000436126	0	0	0	0	0.064	0	0	0.563	0.573
CXCL11	XCL1	9606.ENSP00000306884	9606.ENSP00000356792	0	0	0	0	0.134	0.994	0	0.507	0.997
CXCL11	CXCL9	9606.ENSP00000306884	9606.ENSP00000354901	0	0	0	0.84	0.787	0.994	0.9	0.451	0.999
CXCL13	GZMK	9606.ENSP00000286758	9606.ENSP00000231009	0	0	0	0	0.27	0	0	0.46	0.589
CXCL13	JCHAIN	9606.ENSP00000286758	9606.ENSP00000440066	0	0	0	0	0.173	0	0	0.307	0.402
CXCL13	FDCSP	9606.ENSP00000286758	9606.ENSP00000318437	0	0	0	0	0.436	0	0	0.203	0.531

CXCL13	SAA2	9606.ENSP00000286758	9606.ENSP00000436126	0	0	0	0	0.074	0	0	0.521	0.537
CXCL13	XCL1	9606.ENSP00000286758	9606.ENSP00000356792	0	0	0	0	0.125	0	0	0.524	0.565
CXCL13	CXCL9	9606.ENSP00000286758	9606.ENSP00000354901	0	0	0	0	0.435	0	0.9	0.937	0.996
CXCL9	GZMK	9606.ENSP00000354901	9606.ENSP00000231009	0	0	0	0	0.34	0	0	0.405	0.591
CXCL9	SAA2	9606.ENSP00000354901	9606.ENSP00000436126	0	0	0	0	0.061	0	0	0.558	0.567
CXCL9	XCL1	9606.ENSP00000354901	9606.ENSP00000356792	0	0	0	0	0.204	0.994	0	0.589	0.997
GZMK	JCHAIN	9606.ENSP00000231009	9606.ENSP00000440066	0	0	0	0	0.226	0	0	0.294	0.43
GZMK	XCL1	9606.ENSP00000231009	9606.ENSP00000356792	0	0	0	0	0.274	0.051	0	0.593	0.695
JCHAIN	LYZ	9606.ENSP00000440066	9606.ENSP00000261267	0	0	0	0	0.137	0	0	0.369	0.432
LYZ	MPEG1	9606.ENSP00000261267	9606.ENSP00000354335	0	0	0	0	0.324	0	0	0.57	0.697
