# Supplementary material for: Identification and Preliminary Clinical Validation of Key Extracellular Proteins as the Potential Biomarkers in Hashimoto’s Thyroiditis by Comprehensive Analysis
Source: Biomedicines. 2023 Nov 24;11(12):3127. doi: 10.3390/biomedicines11123127 (PMC10740579; doi:10.3390/biomedicines11123127)
Supplement: Supplementary file 1 [file biomedicines-11-03127-s001.zip › Supplementary Table S2.pdf]

Supplementary Table S2. Characteristics of the study groups.

Patient number	Group	Gender	Age (years)	Pathology of thyroid nodules	Pathology of thyroid tissues	FT3	FT4	TSH	TPOAb	TGAb
1	HT	Female	42	nodular goiter	HT	normal	normal	normal	positive	positive
2	HT	Female	55	follicular adenoma	HT	normal	normal	normal	positive	normal
3	HT	Female	48	nodular goiter	HT	normal	normal	normal	positive	normal
4	HT	Female	37	nodular goiter	HT	normal	normal	normal	normal	normal
5	HT	Female	23	nodular goiter	HT	normal	normal	normal	positive	positive
6	HT	Female	63	nodular goiter	HT	normal	normal	normal	positive	normal
7	NC	Female	29	nodular goiter	normal	normal	normal	normal	normal	normal
8	NC	Female	36	nodular goiter	normal	normal	normal	normal	normal	normal
9	NC	Female	57	nodular goiter	normal	normal	normal	normal	normal	normal
10	NC	Female	45	nodular goiter	normal	normal	normal	normal	normal	normal
11	NC	Female	40	nodular goiter	normal	normal	normal	normal	normal	normal

Abbreviations: HT, Hashimoto's thyroiditis. NC, normal control. FT3, free triiodothyronine. FT4, free thyroxine. TSH, thyroid-stimulating hormone. TPOAb, thyroid peroxidase antibody. TGAb, thyroglobulin antibody.
